# Submicron-Grooved Films Modulate the Directional Alignment and Biological Function of Schwann Cells

**DOI:** 10.3390/jfb14050238

**Published:** 2023-04-23

**Authors:** Zhen Zhang, Yuanliang Lv, Javad Harati, Jianan Song, Ping Du, Peiyan Ou, Jiaqi Liang, Huaiyu Wang, Peng-Yuan Wang

**Affiliations:** 1 Center for Human Tissues and Organs Degeneration, Shenzhen Institute of Advanced Technology, Chinese Academy of Sciences, Shenzhen 518055, China; 2 University of Chinese Academy of Sciences, Beijing 100049, China; 3 Oujiang Laboratory, Key Laboratory of Alzheimer’s Disease of Zhejiang Province, Institute of Aging, Wenzhou Medical University, Wenzhou 325000, China; 4 Shenzhen Key Laboratory of Biomimetic Materials and Cellular Immunomodulation, Shenzhen Institute of Advanced Technology, Chinese Academy of Sciences, Shenzhen 518055, China

**Keywords:** topographical cues, Schwann cells, submicron grooves, cell alignment, peripheral nerve

## Abstract

Topographical cues on material surfaces are crucial for guiding the behavior of nerve cells and facilitating the repair of peripheral nerve defects. Previously, micron-grooved surfaces have shown great potential in controlling nerve cell alignment for studying the behavior and functions of those cells and peripheral nerve regeneration. However, the effects of smaller-sized topographical cues, such as those in the submicron- and nano-scales, on Schwann cell behavior remain poorly understood. In this study, four different submicron-grooved polystyrene films (800/400, 800/100, 400/400, and 400/100) were fabricated to study the behavior, gene expression, and membrane potential of Schwann cells. The results showed that all submicron-grooved films could guide the cell alignment and cytoskeleton in a groove depth-dependent manner. Cell proliferation and cell cycle assays revealed that there was no significant difference between the submicron groove samples and the flat control. However, the submicron grooves can direct the migration of cells and upregulate the expression of critical genes in axon regeneration and myelination (e.g., MBP and Smad6). Finally, the membrane potential of the Schwann cells was significantly altered on the grooved sample. In conclusion, this study sheds light on the role of submicron-grooved patterns in regulating the behavior and function of Schwann cells, which provides unique insights for the development of implants for peripheral nerve regeneration.

## 1. Introduction

Peripheral nerve injury is a challenging issue in neurological diseases, which can pose severe adverse effects on the health and well-being of patients [1,2,3]. Although autologous nerve grafts are considered the gold standard for repairing peripheral nerve injuries [4,5,6], their widespread use in clinical practice is limited due to the scarcity of available nerves, the need for secondary surgeries, and the permanent loss of function in the donor area [7]. In recent years, nerve grafts made from natural or synthetic biomaterials have been increasingly used for peripheral nerve regeneration [8,9]. However, their repairing effect is still insufficient to meet the clinical requirements. To realize better nerve regeneration, numerous scientists are focusing on the modification of the surface properties of biomaterials to facilitate nerve repair. Studies have shown that nerve conduits with electrical conductivity, porosity, appropriate mechanical properties, and appropriate topology can mimic the microenvironment of nerve cells and, thus, promote axonal growth [10]. Among these, the preparation of material surfaces with ordered topographical features (such as grooves, pillars, and pores) using photolithography or soft lithography is widely used due to its ease of operation and non-toxicity [11].

In addition to autologous nerve grafting, the artificial construction of nerve guidance conduits (NGCs) is also a common strategy for peripheral nerve repair. Materials used to construct NGCs need to have good biocompatibility, biodegradability and appropriate mechanical properties [12,13]. NGCs can be divided into natural materials and synthetic materials. Natural materials include collagen, fibrin, gelatin, chitosan, etc. Synthetic materials include poly (ε-caprolactone) (PCL), polylactic acid (PLA), polyglycolic acid (PGA), poly (lactic-co-glycolic acid) (PLGA), etc. However, these materials are biodegradable, where the surface structures gradually disappear with time, resulting in diminished cell–surface interaction. This paper used polystyrene (PS) as the film-forming material to prepare the submicron-grooved surface. PS has good cytocompatibility and adhesion as the raw material of cell tissue culture plates.

It is well known that Schwann cells are glial cells that envelop axons to form myelin sheaths, which are often used as model cells in the studies of peripheral nerve repair. Following peripheral nerve injury, Schwann cells can remove myelin debris and proliferate to form Büngner zone bands, promoting axon regeneration via distal nerve stumps [14]. Moreover, Schwann cells can secrete neurotrophic and growth factors that aid axonal regeneration [15,16,17,18]. Numerous studies have demonstrated that regularly oriented structures can be used to guide the directional alignment and growth of Schwann cells to promote axonal regeneration [19,20,21,22,23,24]. For instance, hierarchically arranged collagen micropatterns were prepared using micro-forming techniques and found to be effective in directing the growth of Schwann cells while upregulating specific genes related to axon regeneration and myelination [25]. Additionally, well-aligned electrospun fibers can promote Schwann cell migration and even neurite growth to repair the dorsal root ganglion [26]. Grooved patterns are another type of topographical cue that can be used to direct the alignment of Schwann cells, in which groove width [27,28], depth [29,30], and geometry [31,32] are key factors influencing cell behaviors.

While earlier studies have focused on the effect of grooves on the morphology of Schwann cells, there are few studies that have systematically examined the effect of grooves on the other behaviors of Schwann cells. Furthermore, to our knowledge, the grooves in most studies are in a micron range [33,34,35], and studies on how submicron grooves regulate the behaviors of Schwann cells have not been reported. More importantly, in these studies, bioactive molecules were used together to enhance the adhesion and bioactivity of Schwann cells on the grooved surface, making it difficult to determine which was the primary factor and impeding the investigation of related biological mechanisms. Therefore, a better understanding of how submicron grooves regulate Schwann cell behaviors and the underlying mechanisms is crucial at the present stage.

In this study, we prepared four diverse sizes of submicron-grooved PS films and systematically studied their effects on the properties of Schwann cells, including cell adhesion, morphology, proliferation, migration, transcriptome profile, and membrane potential. The relationship between the size of the submicron grooves and the behaviors of the Schwann cells was explored. Our findings provide valuable insights for understanding cell–pattern interactions and the preparation of advanced NGCs for peripheral nerve injury.

## 2. Materials and Methods

### 2.1. Fabrication of Submicron-Grooved Films

To produce topographical patterns on polystyrene (PS) films, we utilized four distinct sizes of grooved silicon substrates with the groove width/depth (in nanometers) of 800/400, 800/100, 400/400, and 400/100, according to our previous study [36]. Firstly, we mixed a 10:1 ratio of polydimethylsiloxane (PDMS) to curing agent thoroughly to create silicon rubber molds. The mixture was then poured onto the grooved silicon substrate and vacuumed to eliminate air bubbles. The molds were then heated and fixed in an oven at 80 °C for 3 h, after which they could be easily demolded from the grooved silicon substrates, obtaining PDMS molds with submicron-grooved topography. We also prepared PDMS molds on a flat silicon substrate as the control group.

A polyethylene terephthalate (PET) plate of 2 cm × 2 cm was utilized as the substrate to produce submicron-grooved PS films. Subsequently, a PS in toluene solution (5 wt%) was added dropwisely onto the PET plate. The patterned side of the PDMS mold was then inverted onto the PS solution, and pressure was added onto the back of the mold to ensure uniformity. The PS films with submicron grooves were dried naturally overnight and then cut into 1 cm × 1 cm squares for the subsequent experiments.

### 2.2. Characterization of Submicron-Grooved Films

The integrity of the submicron-grooved topography was assessed by observing the morphology of the PS submicron-grooved films using a scanning electron microscope (SEM, Carl Zeiss Supra 55, Jena, Germany). Additionally, as the hydrophilicity of the substrate surface is a critical factor for cell behaviors, we measured the water contact angle of the PS submicron-grooved films with or without air plasma treatment (0.4 mbar, 200 W and 3 min) using a contact angle measurement instrument (Theta Lite, Biolin, Espoo, Finland), with three parallel samples being used for the measurement of each group.

### 2.3. Cell Culture

The immortalized human-derived Schwann cells obtained from plexiform neurofibroma (ATCC, CRL-3390, Lot Number: 70024530) were purchased from the Cell Bank, Chinese Academy of Sciences (Shanghai, China). The Schwann cells were cultured in high-glucose Dulbecco’s modified Eagle’s medium (DMEM, Hyclone, Logan, UT, USA) and supplemented with 10% (*v*/*v*) fetal bovine serum (FBS, Gibco, NSW, Australia) and 1% antibiotics of penicillin/streptomycin (100 units/mL of penicillin and 100 mg/mL of streptomycin, Hyclone, Logan, UT, USA).

### 2.4. Cell Viability

The submicron-grooved films with various sizes were placed in 24-well plates, cleaned by air plasma for 3 min, sterilized with 75% ethanol, and then washed three times with PBS before cell seeding. For the cell proliferation assay, Schwann cells were seeded in 24-well plates at a density of 3 × 10^4^ cells/mL. After 1, 3, and 5 days of cell culture, the medium was removed, and cells on the samples were washed thrice with PBS. Next, 10% Cell Counting Kit-8 reagent (CCK-8, Beyotime, Shanghai, China) was added and incubated at 37 °C for another 4 h, and 150 µL of supernatant was removed from each well and transferred to a 96-well plate. Finally, the absorbance value of the supernatant was measured at 450 nm using a SpectraMax M5 microplate spectrophotometer (Molecular Devices, San Jose, CA, USA). The absorbance value determined from the flat group was used as control to calculate the cell viability.

Live–dead staining was also performed to determine the viability of Schwann cells on submicron-grooved films. After 3 days of cell culture, the medium was removed, and the cells were stained with 2 μM Calcein-AM (Beyotime) and 4.5 μM propidium iodide (PI, Beyotime) for 15 min at 37 °C. The stained samples were then imaged using a fluorescence microscope (Olympus, Tokyo, Japan), while the live cells were identified by green fluorescence and the dead cells were identified by red fluorescence.

### 2.5. Cell Morphology

After 24 h of cell culture, the morphological features of Schwann cells cultured on submicron-grooved films were evaluated. The overall and individual morphological characteristics of Schwann cells on different samples were observed using optical and scanning electron microscopes (SEM), respectively. Thirty cells were randomly selected from different groups based on SEM images, and their cell area, and aspect ratio and orientation angle parameters were quantified using ImageJ software (v1.8.0).

### 2.6. Cell Adhesion

To evaluate the cell adhesion of Schwann cells on the submicron-grooved films, the Schwann cells cultured on different samples for 24 h were fixed using 4% paraformaldehyde for 20 min and permeabilized with 0.2% Triton X-100 in PBS for 10 min. After being washed thrice with PBS, the cells were blocked with 3% bovine serum albumin (BSA) at 37 °C for 1 h. Next, a primary antibody against vinculin (ab129002; Abcam, Cambridge, UK) was added at a dilution of 1:250 in 1% BSA and incubated at 4 °C overnight, followed by incubation with a secondary antibody of Alexa Fluor 488 for 3 h. In addition, Rhodamine 555 phalloidin (1:200) and DAPI (1:1000) were used to stain the cytoskeleton and nuclei of cells for 30 min. Finally, the stained samples were visualized and imaged using a Zeiss LSM-710 laser-scanning confocal microscope (LSM-710, Zeiss, Jena, Germany).

### 2.7. Cell Migration

The migration trajectory of Schwann cells on submicron-grooved films was recorded using the JuLI Stage Living cell monitoring system (Nano Entek, Seoul, South Korea) equipped with an incubator (37 °C, 5% CO_2_). Cells were seeded onto the submicron grooves at a density of 3 × 10^4^ cells/mL and allowed to adhere for 1 h before being transferred to the monitoring incubator. The movement of Schwann cells on the submicron grooves was recorded at 1 h intervals for 24 h.

The images of individual cells were manually tracked and analyzed using ImageJ software to obtain a series of X/Y coordinates corresponding to a time interval of 1 h. The original position of each cell was defined as (0, 0), and the trajectory of cells in the X/Y plane during the 24 h was reconstructed. Thirty cells were randomly selected from each group to record the trajectories.

### 2.8. Cell Cycle Analysis

Flow cytometry (BD Bioscience, San Jose, CA, USA) was utilized to evaluate the cell cycle progression of Schwann cells on submicron-grooved films. After 5 days of culturing on various samples, the Schwann cells were digested and collected in centrifuge tubes, which were then centrifuged at 1000× *g* for 5 min. Subsequently, the cells were fixed overnight by adding 70% ethanol pre-cooled in an ice bath. Next, the cells were rinsed with pre-cooled PBS and slowly resuspended in a prepared propidium iodide staining solution, which was then subjected to a 30 min incubation at 37 °C in darkness. Finally, the cell precipitate was filtered to obtain single cells, and the flow assay was completed within 1 h. ModFit LT5.0 analysis software was employed to analyze the cellular DNA content.

### 2.9. RNA Sequencing

The transcriptome sequencing was conducted according to the manufacturer’s instructions (OE Biotech Co., Ltd., Shanghai, China). Total RNA from Schwann cells cultured on samples for 5 days was extracted using TRIzol reagent (Invitrogen, Carlsbad, NM, USA) and stored in liquid nitrogen. The RNA integrity was evaluated using an Agilent 2100 Bioanalyzer (Agilent Technologies, Santa Clara, CA, USA). The VAHTS Universal V5 RNA-seq Library Prep Kit (Vazyme, Nanjing, China) was used to construct transcriptome libraries per the manufacturer’s instructions. The libraries were sequenced using the Illumina Novaseq 6000 sequencing platform to obtain read counts for each sample. Differentially expressed genes (DEGs, with a *q* value < 0.05 and fold change > 2) were analyzed using the DESeq2 software. Enrichment analysis of DEGs was performed using GO Reactome and KEGG based on the hypergeometric distribution.

### 2.10. Gene Expression

After culturing Schwann cells on various submicron-grooved films for 5 days, real-time quantitative polymerase chain reaction (RT-qPCR) was used to assess the expression levels of target genes. Total RNA was extracted from Schwann cells using TRIzol reagent (Invitrogen, Carlsbad, NM, USA), and 1 μg of total RNA was reverse transcribed to cDNA using Superscript III reverse transcriptase with random hexamer primers (RR037A, Takara, Beijing, China). RT-qPCR was performed on a real-time fluorescence quantitative PCR instrument (LightCycler 96, Roche, Basel, Switzerland) using the following amplification parameters: 95 °C for 2 min, 40 cycles at 95 °C for 15 s, 60 °C for 15 s, and 72 °C for 30 s. The primers of target genes (Table 1) were synthesized by Guangzhou IGE Biotechnology Ltd., and GAPDH was used as the housekeeping gene for normalization. The fold changes in the expression of each target gene were compared by calculating 2^−ΔΔCt^.

### 2.11. Cell Membrane Potential

To record the membrane potential of cells on different samples, electrophysiological experiments were conducted using a HEKA EPC10 amplifier (HEKA Elektronik GmbH, Lambrecht, Goettingen, Germany) in the current clamp recording mode. After being cultured on submicron-grooved (800/400) and flat films for 3 days, Schwann cells were perfused with a standard extracellular solution containing 140 mM NaCl, 3 mM KCl, 1.5 mM MgCl_2_, 2 mM CaCl_2_, 10 mM HEPES, and 10 mM Glucose. The pH of the solution was adjusted to 7.4 with NaOH, and the osmolarity was between 300–320 mOsm. The internal solution contained 164 mM KCl, 2 mM MgCl_2_, 1 mM CaCl_2_, 10 mM HEPES, 11 mM EGTA, and 10 mM glucose, with a pH of 7.3 adjusted with KOH. All the electrophysiological experiments were performed at room temperature.

### 2.12. Statistical Analysis

The significant differences between groups were evaluated using ANOVA, with one-way ANOVA analysis performed using GraphPad Prism 8 software. The results are presented as the mean ± standard deviation (SD) of 3 independent experiments. A *p* value less than 0.05 was considered to be statistically significant.

## 3. Results

### 3.1. Characterization of Submicron-Grooved Films

**Scheme 1:**
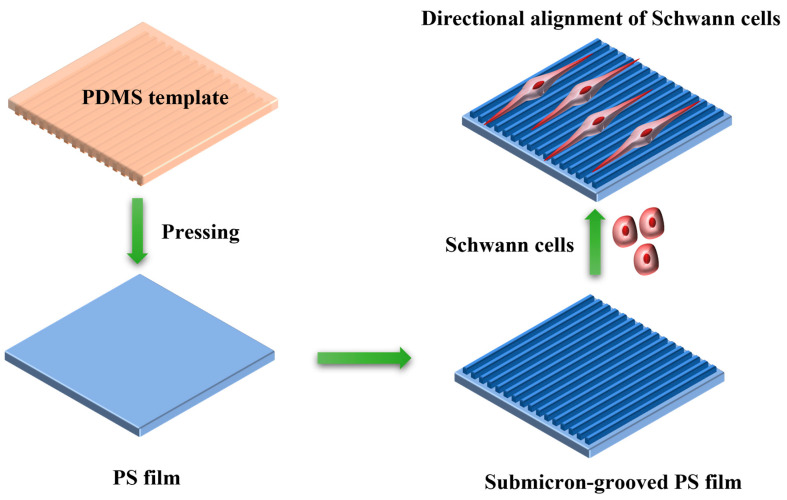
A schematic illustration showing the fabrication process of submicron-grooved films, which can be utilized to direct the alignment of Schwann cells.

**Figure 1:**
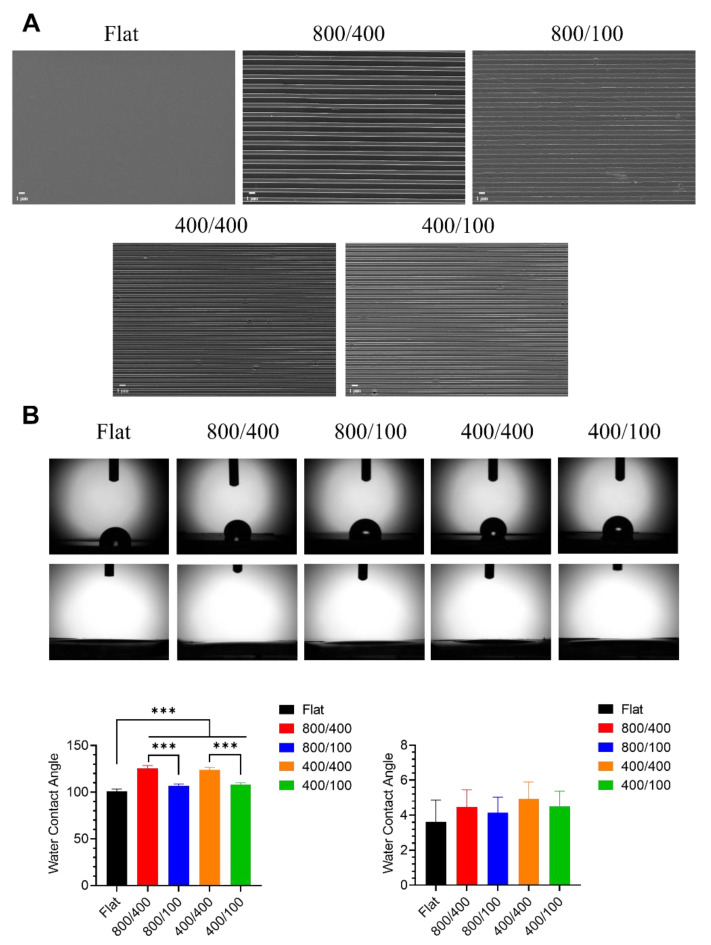
(**A**) Surface morphology of different submicron-grooved and flat films observed by SEM; scale bars are 1 µm. (**B**) Water contact angles detected on different samples without (above) or with (below) air plasma treatment and the corresponding statistical analysis. *** represent *p* < 0.001, respectively (n = 3).

Soft lithography was used to prepare grooved PS patterns. Four PDMS stamps with submicron groove structures were made from the master silicon wafers. A PS solution was dropped on a PET substrate and dried under the PDMS stamps (Scheme 1). The structural integrity of the grooves is crucial for the reliability of the cell culture platform. In this respect, SEM was utilized to assess the surface geometry of various submicron-grooved films. As show in Figure 1A, the submicron grooves possess a complete and uniform structure, with pronounced ridges and furrows typical of grating structures. The different light reflection from the submicron-grooved films leads to differences in brightness and darkness in the background, where the shallower grooves exhibit a lower contrast, whereas the deeper grooves show a higher contrast for the same width.

Surface wettability is another critical factor affecting cell adhesion. Therefore, water contact angles are measured on different submicron-grooved films with or without air plasma treatment using a contact angle measurement instrument. As depicted in Figure 1B,C, all the samples exhibit an explicit hydrophobicity with water contact angles greater than 90°. Notably, the water contact angle measured on the submicron-grooved films is considerably larger than that determined on the flat films. With increased groove depth, the water contact angle progressively rises to approximately 110° (800/100 and 400/100) and 120° (800/400 and 400/400). After plasma treatment, the grooved patterns became superhydrophilic due to a lateral capillary force inside the grooves, which is consistent with a previous study [37].

### 3.2. Cell Viability

**Figure 2:**
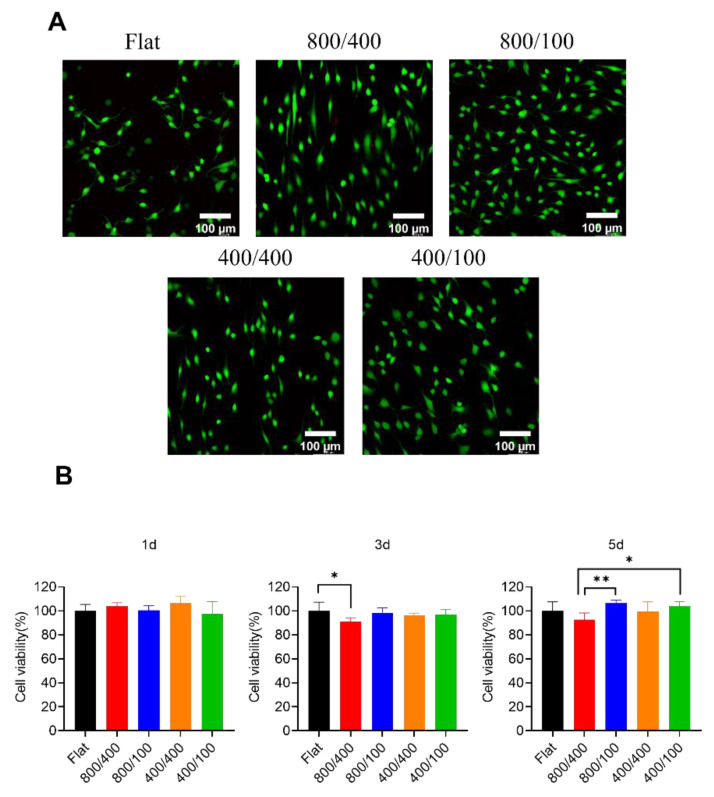
(**A**) Live–dead staining images of Schwann cells on different samples; scale bars are 100 µm. (**B**) Cell viability of Schwann cells cultured on different samples for 1, 3, and 5 days. * and ** represent *p* < 0.05, *p* < 0.01, respectively.

Both live–dead staining and CCK-8 assays were performed to evaluate the viability of Schwann cells cultured on different samples. After 2 days of culturing on different submicron-grooved films, Schwann cells were stained with Calcein-AM and PI. Figure 2A shows the images of live–dead stained cells, and no significant difference can be found between the different groups. The abundant green fluorescence of viable cells demonstrates the excellent cytocompatibility of the groove samples. The CCK-8 assay evaluated the proliferation of the Schwann cells after 1, 3, and 5 days of culturing on the four samples. The proliferation analysis of the Schwann cells, shown in Figure 2B, indicates a minimal variation between the grooved and flat films at different cell culture times, and only the cell viability determined on the 800/400 grooved film is slightly lower than the other groups at day 5.

### 3.3. Cell Morphology

**Figure 3:**
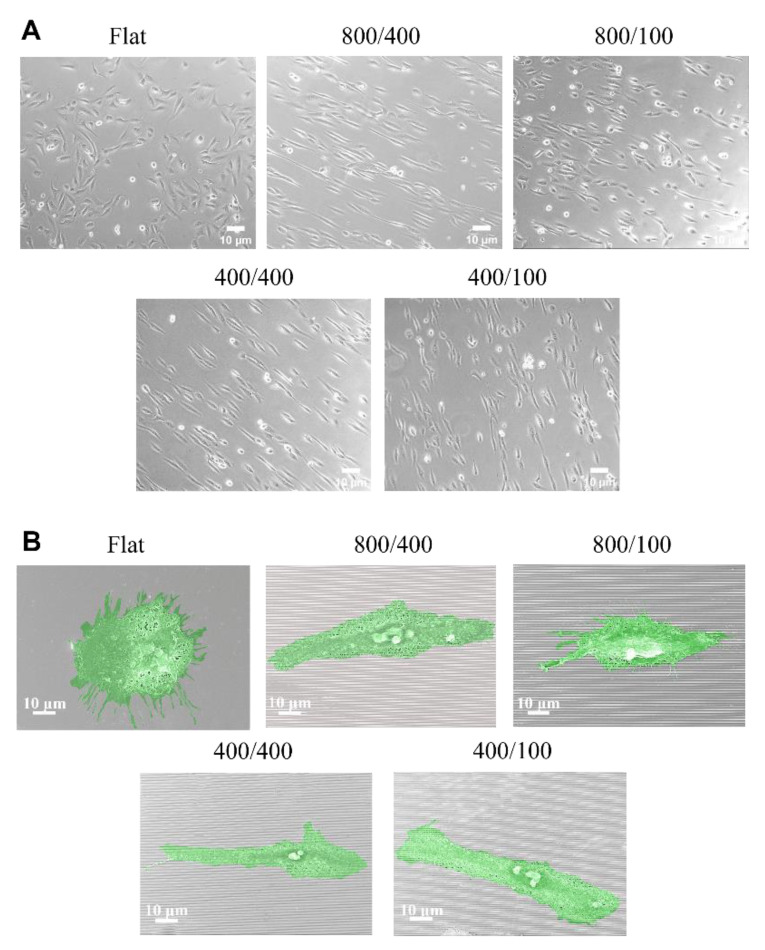
Cell morphology of Schwann cells cultured on different samples: (**A**) Optical microscope photographs and (**B**) SEM photographs. Scale bars are 10 µm.

The impact of a submicron-grooved structure on cell morphology is primarily reflected in the directional arrangement of the Schwann cells. To evaluate the cell morphology, the cells were observed and analyzed after 24 h by optical microscopy and SEM. As shown in Figure 3A, the cells adhere randomly on the flat surfaces with circular or triangular cell shapes. However, on the grooved films, the cells show a directional arrangement, elongated in a linear pattern and growing in the same direction. In more detail, the degree of the directional alignment of the Schwann cells on different films follows the order of 800/400 > 400/400 > 400/100 > 800/100 > Flat. The SEM images shown in Figure 3B confirm that Schwann cells can span several ridges. The filopodia were located mainly in the two ends of the cells. Conversely, on the flat film, the cells had many filopodia and extensions surrounding the cells.

**Figure 4:**
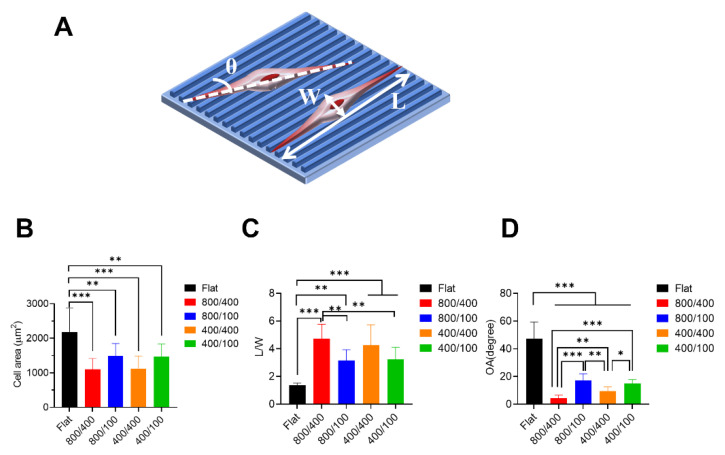
(**A**) A schematic illustration showing the calculation method of the length (L)-to-width (W) ratio and the orientation angle (θ) of Schwann cells; (**B**) cell area of Schwann cells cultured on different samples; (**C**) L/W ratio of Schwann cells cultured on different samples; (**D**) orientation angle (OA) of Schwann cells cultured on different samples. *, **, and *** represent *p* < 0.05, *p* < 0.01, and *p* < 0.001, respectively (n = 30).

Other cell morphological indicators including area, aspect ratio or elongation (length/width, L/W), and orientation angle (OA) are also evaluated to assess the impact of different submicron grooves (Figure 4A). Figure 4B shows that the Schwann cells spread well on flat film, resulting in a significantly larger cell area than those cultured on the submicron-grooved samples. Among the samples with grooved structures, no significant difference in cell area can be observed. The cell elongation showed that the Schwann cells cultured on submicron grooves exhibit a significantly greater L/W value than those on flat film (Figure 4C). Moreover, the L/W values of cells on 800/400 grooves are significantly higher than those on 800/100 and 400/100 grooves. As direct evidence of cell arrangement, the orientation angles of all the cells on the different submicron grooves are below 20°, compared to the random orientation of cells on flat film (Figure 4D). The different grooves pose different effects on the orientation of Schwann cells, with the lowest orientation angle (the highest alignment of cells) being observed on the 800/400 grooves.

### 3.4. Cell Adhesion and Cytoskeleton

**Figure 5:**
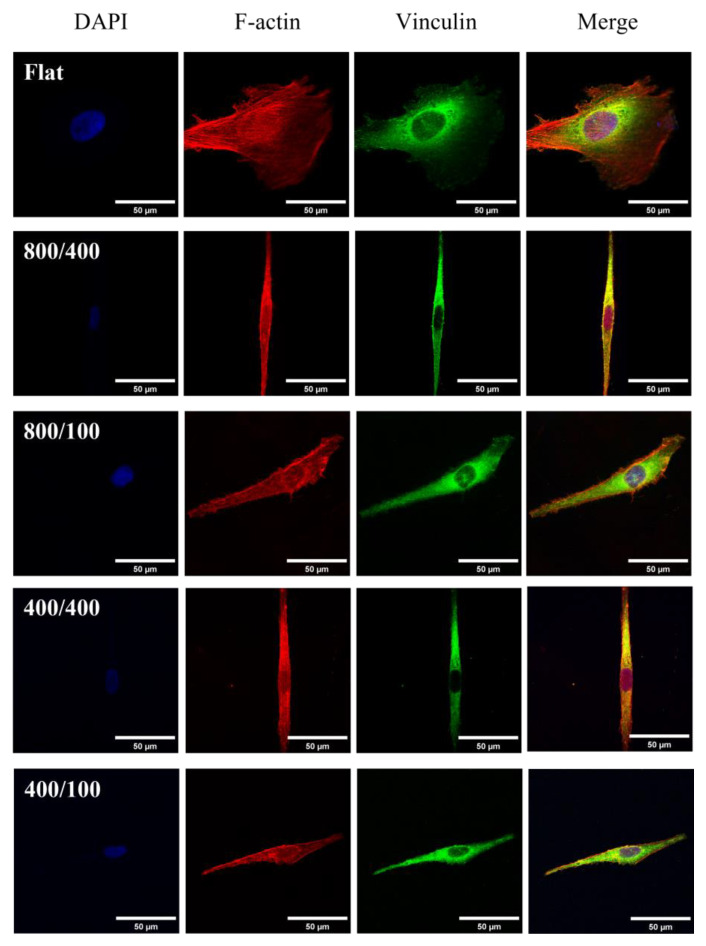
Immunofluorescent staining of Schwann cells cultured on different samples by laser confocal microscopy. The nuclei, F-actin, and vinculin were stained with DAPI (blue), phalloidin (red), and primary antibodies of anti-vinculin (green), respectively. Scale bars are 50 µm.

In a next step, the effect of submicron grooves on the adhesion and skeleton of Schwann cells is evaluated by immunofluorescence staining. After the Schwann cells adhered to the submicron-grooved films for 24 h, the cells were immunofluorescence-stained with phalloidin and vinculin antibodies and observed under a laser confocal microscope. As shown in Figure 5, the F-actin microfilament structure of the Schwann cells forms bundles that elongate along the grooves, which is quite different from that observed on the flat film. Moreover, vinculin is evenly distributed at both ends of the elongated cells on the grooves, whereas it is primarily located around the nuclei of the Schwann cells cultured on the flat surface. These findings suggest that the groove structure can reshape the cytoskeletal structure and influence the distribution of adhesion proteins.

### 3.5. Cell Migration

**Figure 6:**
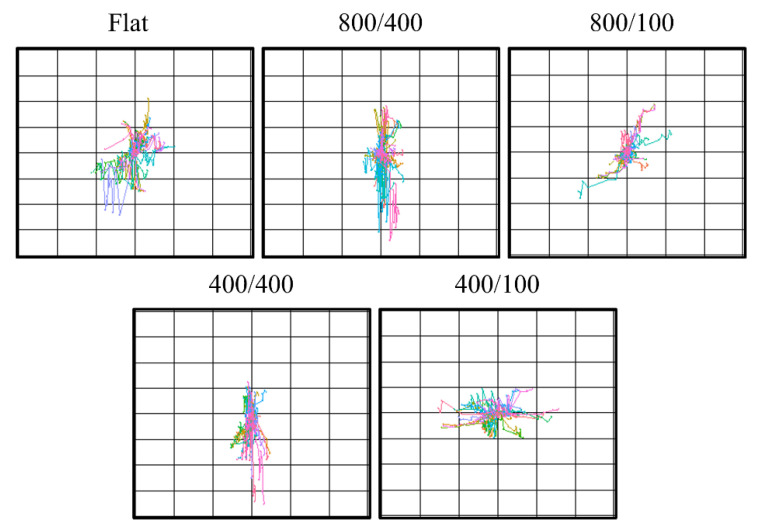
The migration traces of Schwann cells on different samples within 24 h.

To evaluate the cell migration, cell positions were captured every hour within 24 h after cells seeding on different samples. The cell movement trajectory is displayed in the X/Y axis coordinate plot (Figure 6), with the origin (0, 0) as the starting point. The results show that most of the Schwann cells migrate linearly along the Y-axis direction on the 800/400 and 400/400 grooves, and the movement of the Schwann cells is relatively less directional on the 400/100 and 800/100 grooves. In contrast, the movement of the Schwann cells on the flat surface was non-directional. These findings suggest that the depth of the grooves plays an essential role in directing the migration of Schwann cells.

### 3.6. Cell Cycle Analysis

**Figure 7:**
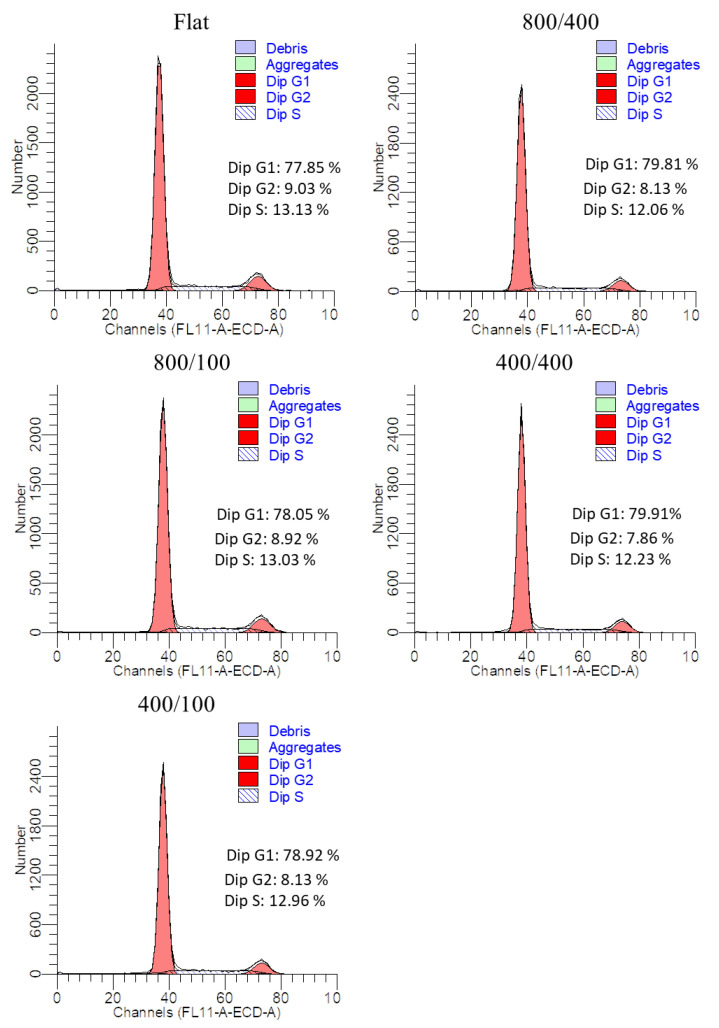
The cell cycle of Schwann cells after being cultured on different samples for 5 days.

After 5 days of culturing, the Schwann cells cultured on different samples were stained with PI. Then, the cell cycle was analyzed using flow cytometry. As depicted in Figure 7, the G1 phase is predominant for the Schwann cells cultured on both the grooved and flat samples, with no noticeable differences among them. The highest percentage of Schwann cells in the G2 phase can be observed on the flat sample (9.03%), followed by the 800/100 grooves (8.92%), while minimal differences can be observed among the 800/400, 400/400, and 400/100 groups (7.86–8.13%). The S phase is the DNA synthesis phase of cells. According to the results, the percentage of Schwann cells in the S phase is slightly higher in the flat (13.13%), 800/100 (13.03%), and 400/100 (12.96%) groups compared to the 800/400 (12.06%) and 400/400 (12.23%) groups. Our findings suggest that the depth relative to the width of the grooves has a slight effect on the cell cycle of Schwann cells.

### 3.7. Gene Expression

It is demonstrated by the aforementioned results that the Schwann cells exhibit the most conspicuous cell alignment behavior on the 800/400 groove among the various groups. Thus, RNA sequencing were performed on the 800/400 and flat films for 5 days. Trizol was used to collect total RNA from the cells cultured on different films after 5 days, and the gene expression was analyzed using an Illumina platform.

**Figure 8:**
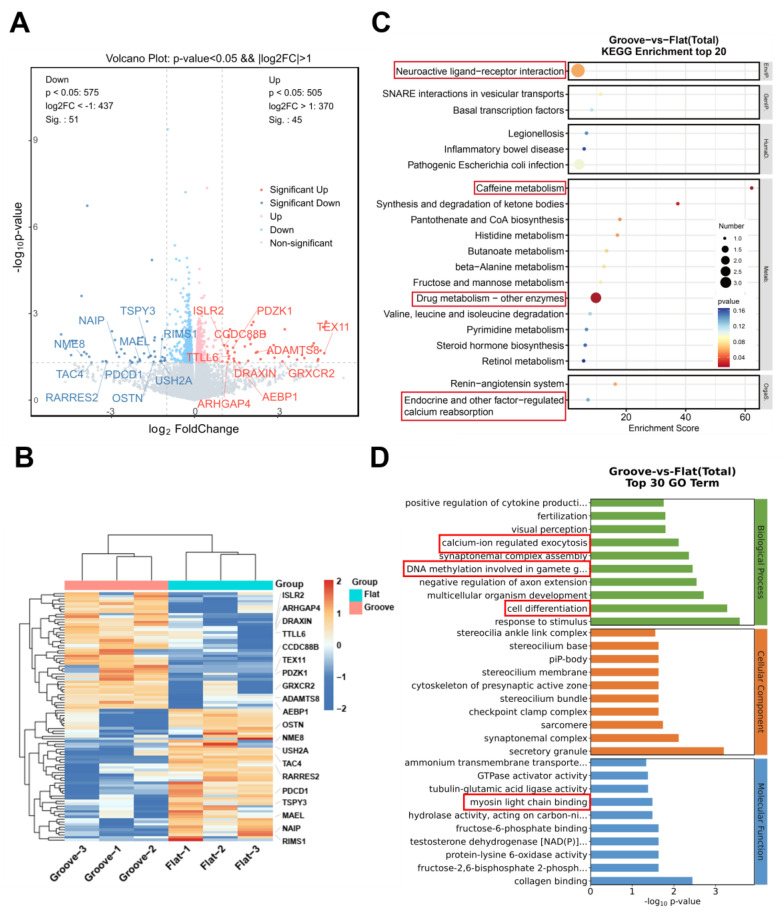
Transcriptome comparison of Schwann cells after being cultured on submicron-grooved (800/400) and flat films for 5 days (n = 3): (**A**) Volcano plots the total number of genes identified by RNA sequencing. (**B**) The heat map represents the differentially expressed genes. DEGs were defined as |log2(fold change)| > 1 and *q* value < 0.05. (**C**)The KEGG pathway enrichment of DEGs under submicron-grooved and flat films conditions. (**D**) Gene Ontology (GO) enrichment analysis of DEGs in both cases.

The outcomes illustrated in Figure 8A,B reveal a total of 96 differently expressed genes (DEGs) between the 800/400 and the flat films, of which 45 genes are upregulated and 51 genes are downregulated. The upregulated DEGs are mainly associated with axon guidance, synaptic complex assembly, extracellular matrix secretion, cytoskeleton, and protein localization and transport, whereas the downregulated DEGs are primarily related to cell differentiation, cell membrane potential regulation, immune regulation, inflammatory response, and cell spreading. In addition, KEGG pathway analysis and GO enrichment were conducted to gain further insight into the functional annotations of the DEGs in both cases. Notably, the findings indicated that pathways influencing axon extension were predominantly enriched in the submicron-grooved group compared to the flat group, including the negative regulation of axon extension, neuroactive ligand–receptor interaction, calcium-ion-regulated exocytosis, etc.

**Figure 9:**
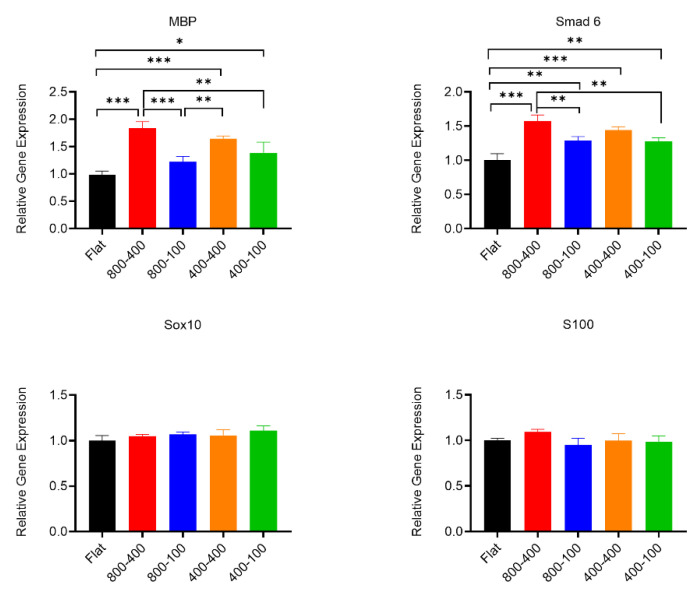
Relative gene expression associated with specific markers of Schwann cells after being cultured on different samples for 5 days. *, **, and *** represent *p* < 0.05, *p* < 0.01, and *p* < 0.001, respectively (n = 3).

Afterwards, the expression of specific genes of the Schwann cells on all grooved patterns is determined by RT-qPCR. Figure 9 shows the gene expression of Schwann cells in different groups after 5 days of cultivation. The results demonstrate that the submicron grooves can upregulate the expression of MBP and Smad6 in Schwann cells, while their effect on the expression of Sox10 and S100 is negligible. Similarly to the previous findings, the MBP and Smad6 expressions are mainly affected by the depth of the grooves as a significantly higher expression of these two genes can be detected in the 800/400 and 400/400 groove groups, with the 800/400 groove group demonstrating the most pronounced effect.

### 3.8. Cell Membrane Potential

**Figure 10:**
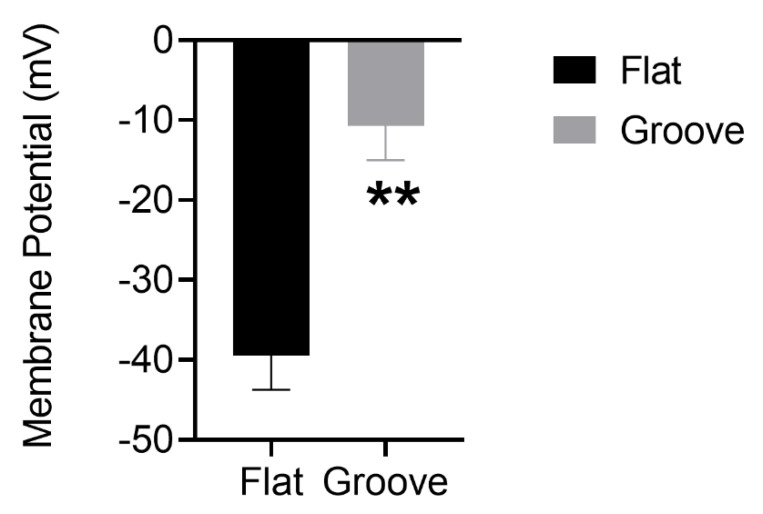
Membrane potential of Schwann cells after being cultured on submicron-grooved (800/400) and flat films for 3 days. ** represent *p* < 0.01 (n = 5).

After 3 days of culturing, the patch clamp measurement is utilized to investigate the disparity in the membrane potential of the Schwann cells cultured on the 800/400 groove and flat films. As illustrated in Figure 10, the membrane potential of the Schwann cells cultured on the 800/400 groove is approximately −10 mV, while the value determined from the flat film group is approximately −40 mV. These findings imply that submicron grooves can considerably alter the membrane potential of cultured Schwann cells, which may be attributed to their effect on cell morphology and orientation.

## 4. Discussion

Peripheral nerve injury is a prevalent condition caused by accidents or natural disasters, which causes over one million people to suffer severe physical and psychological injuries annually [38,39,40]. Although peripheral nerves are self-repairable, such ability is limited for the repair of distant nerve damage (typically greater than 5 mm) [41]. Autologous nerve grafting, the gold standard for repairing peripheral nerve injury, is limited for clinical application due to the source limitations, secondary surgery, and permanent damage to the donor area [25,42]. Nerve conduits constructed by materials with surface topography have shown great potential in peripheral nerve repair as they can guide the growth of nerve protrusions and axonal extension [43,44]. Several studies have demonstrated that well-aligned groove structures can provide physical guidance and promote the alignment of Schwann cells [45]. However, most of the previous studies have focused on the effect of micron-sized grooves on the behaviors of Schwann cells [21,46]. In this study, we investigated the cellular behaviors, such as adhesion, proliferation, and migration, of Schwann cells on submicron grooves with different sizes. 

Furthermore, the effects of different grooves on cell cycle, cell membrane potential, and gene expression profiles were investigated, aiming to better understand the biological functions and potential mechanisms of submicron grooves in regulating Schwann cells. Our experimental results show that Schwann cells exhibit significant directional growth and migration on the submicron-grooved films compared to the random orientation of the flat film and that this directional guidance effect was closely related to the depth of the grooves. Furthermore, the submicron groove structure elongates Schwann cells, resulting in restricted cell spreading. Thus, the area of the Schwann cells on the submicron grooves is much smaller than on the flat films. However, the cell viability and cell cycle of Schwann cells did not differ significantly between the grooved and flat surfaces. In addition, the submicron-grooved films significantly upregulated the expression of genes related to axonal regeneration and myelination relative to flat films. Finally, the submicron grooves could also substantially affect cell membrane potential compared to the flat surfaces.

In our study, submicron-grooved films were prepared using soft lithography, which offers advantages such as ease of preparation, high efficiency, and reusability [47,48]. Biocompatible and biodegradable polymers such as polylactide-poly (PCL) [46], polypropylene-glycolic acid copolymer (PLGA) [29], polylactic acid (PLA) [14], gelatin [49], and chitosan [33] are commonly used in peripheral nerve repair studies. Among them, PCL is one of the most used synthetic polymers to manufacture NGCs. PCL is an FDA-approved material with excellent mechanical properties and ease of processing [50]. Additionally, collagen, as a natural polymer, also has good biocompatibility and, thus, is widely used for axonal regeneration [51,52]. In this study, Polystyrene (PS) was used to prepare the submicron-grooved films because it provides good biocompatibility, excellent film-forming ability, and water stability, ensuring the formation of stable and precise groove structures. The SEM images reveal that the prepared submicron-grooved films have a well-defined ridge/furrow structure essential for the aligning and growth of Schwann cells. It is well known that the surface wettability of biomaterials is critical for the adhesion and proliferation of mammalian cells [53]. The results show that all the submicron-grooved films, as well as the flat film, are changed from hydrophobic to hydrophilic after plasma treatment, which is favorable for cell adhesion.

The morphology and orientation of cells are the most intuitive phenomena of cells cultured on biomaterials. Several previous studies have indicated that biomaterials with groove structures can direct the alignment and elongation of stem cells [49,54,55]. Nevertheless, most of the previous studies contribute to the optimization of groove dimensions (such as depth, width, and spacing) in the micron scale. To the best of our knowledge, this study is the first attempt to investigate the effect of submicron grooves on Schwann cell behaviors. Specifically, it is observed that submicron grooves can modulate the directional orientation of Schwann cells within 1 day, in which most of the cells are elongated and aligned in the same direction of the submicron grooves. In contrast, the cells cultured on the flat film are randomly distributed without orientation. A further SEM analysis reveals that Schwann cells, which have a size of approximately 10 µm, can span many submicron ridges and furrows. Interestingly, we found that the depth of the groove is a crucial factor influencing the orientation arrangement of Schwann cells on submicron grooves. Specifically, our results show that the degree of orientation of the Schwann cells increases with the depth of the groove, which is consistent with a previous study [29]. However, in contrast to our findings, some other studies have demonstrated groove width as the most critical parameter influencing the arrangement of Schwann cells [56]. This difference may be due to the varied sizes of grooves used in different studies. In our study, we selected submicron grooves over micron ones and found that the effect of groove depth on Schwann cell morphology is more pronounced than that of groove width. Compared to the flat film, submicron grooves can significantly reduce cell area, increase aspect ratio, and decrease the orientation angle of Schwann cells. These observations suggest that submicron groove structures may inhibit cell spreading. 

These findings indicate that Schwann cells can rapidly respond to the structures of submicron grooves and undergo morphological changes. It is noteworthy that our findings are contradictory to the previous notion that only the groove structures with dimensions close to cells can be perceived by cells [57]. We propose that the effect of submicron grooves in guiding Schwann cell arrangement relies on the response of cytoskeleton and vinculin proteins. Immunofluorescence staining confirms that the groove structures can reshape the distribution of F-actin and vinculin along the groove direction, and the cytoskeleton and nucleus are significantly elongated by the guidance of the submicron grooves. Taken together, our results demonstrate that submicron grooves can regulate the morphology and arrangement of cultured Schwann cells, with groove depth being a more critical factor than groove width.

Generally, the alternation in cell morphology may impact the biological functions of cells. In our study, the outcomes of CCK8 indicate that the grooves do not compromise the proliferation of Schwann cells, which contrasts the results of previous studies that suggest that groove structures are unfavorable for cell proliferation [58,59]. This difference may be attributed to the relatively small size of grooves employed in our study, leading to stronger cell–groove contact than the boundary effects [30]. The cell cycle analysis can reflect intracellular DNA synthesis, which is closely related to cell proliferation [60]. Our results demonstrate that there is no significant difference of the cell cycle of Schwann cells among different groups, which is consistent with the results of cell proliferation. 

The migration of Schwann cells also plays a crucial role in neural repair [61,62]. Consequently, we investigated the migration behavior of Schwann cells on submicron grooves and found that most Schwann cells maintain linear movement in the same direction of submicron grooves, whereas the movement of Schwann cells on flat film is random. This difference may be related to the traction force provided by F-actin in the cytoskeleton. The groove structures can reshape the cytoskeleton arrangement, and, thereby, enough traction force is contributed to the directional migration of the Schwann cells on the grooves [63]. Interestingly, the directional migration of the Schwann cells is closely associated with the depth of the grooves, with more Schwann cells migrating directionally along the grooves as the depth of the grooves increases. These results indicate that the groove depth can not only affect the morphology of Schwann cells but also provide a pronounced effect on cell migration.

To gain a better understanding of the regulatory role of groove structure on Schwann cell behaviors, we further investigated the effect of submicron grooves on the gene expression of Schwann cells using RNA sequencing and RT-qPCR. Ninety-six DEGs were identified by high-throughput screening. Subsequent GO enrichment analysis revealed that the genes associated with axon guidance, synaptonemal complex assembly, extracellular matrix secretion, cytoskeleton, and protein localization and transport are upregulated. In contrast, the genes related to cell differentiation, membrane potential regulation, immune response regulation, inflammatory response, and substrate adhesion-dependent cell spreading are downregulated. The gene expressions of the specific markers (MBP, Smad6, S100, and Sox10) in Schwann cells were also evaluated. Our results indicate that the submicron grooves can significantly increase the levels of Smad6 and MBP genes. Smad6 is a crucial axonal membrane protein that aids neuronal plasticity and regeneration [64]. The upregulation of Smad6 gene is beneficial for axonal growth. Similarly, MBP is essential for Schwann cells to form myelin around nerve axons [65,66]. An increase in the gene level of MBP suggests that Schwann cells are beginning to form myelin around nerve axons [67]. Taken together, our findings demonstrate that submicron grooves are favorable to axon regeneration and myelination, which is critical for peripheral nerve regeneration.

The alternation of cell morphology and cytoskeletons can possibly impact the membrane potential of cells. In this regard, we assessed the membrane potential of Schwann cells cultured on submicron-grooved and flat films. Our results demonstrate that the submicron groove structures can significantly alter the membrane potential of Schwann cells, indicating the remarkable change in the membrane proteins [68,69]. Additionally, micro-patterns have been found to interact with cell surface proteins, triggering a series of cascade reactions within cells [70]. This interaction could lead to changes in the expression of receptors related to membrane potential in Schwann cells, including sodium and potassium receptors, resulting in alterations in membrane potential. These findings verify that the submicron grooves substantially affect the behaviors of Schwann cells.

Normal nerve tissue is distributed in long strips with good directional growth characteristics, and simulating this structure will facilitate nerve regeneration. Previous reports have stated that ideal nerve guidance conduits must have the structural characteristics of longitudinally aligned regenerating axons [71,72]. Surfaces with nanoscale gratings, microgrooves, and channels are commonly used as substrates in neural differentiation studies because they mimic the natural neuronal and glial cell microenvironment in vivo [73,74], thus facilitating axon regeneration and development. In this paper, we use submicron groove structures to provide a linear 3D-like environment for Schwann cells. From a physiological perspective, submicron grooves can mimic and guide the directional growth and migration of Schwann cells in vivo, reducing axonal dispersion and mistargeted regeneration. The expression of key genes for axonal regeneration and myelination upregulated by submicron-grooved films could illustrate their potential application in peripheral nerve regeneration.

The in vitro construction of artificial nerve guidance conduits (NGCs) is currently the most common method for peripheral nerve repair. Many studies have reported that incorporating micro- and nano-scale filler materials into NGCs can promote axonal regeneration. These fillers include microlumen/microchannel structures, neatly aligned electrospun fibers, hydrogel matrices, and conduits rolled up by material sheets. In vivo, collagen–chitosan (CCH) scaffolds with longitudinal, parallel microchannels showed nerve regeneration and functional recovery similar to autografts when used to repair 15 mm sciatic nerve defects in rats. This micropatterning affects the migration and axonal regeneration of Schwann cells at the microscopic level of these cellular interactions [75]. Interestingly, many studies have used rolled up micro- or nanopatterned 2D surfaces, or patterned micro- or nanomaterials as filler materials for NGCs. For example, the surface of self-rolling silicon nitride microtubule arrays can provide growth channels and accelerate neuronal cell growth [76]. Another study also showed that micron-sized grooved surfaces can promote the development and directed axonal growth of adult neural stem cells and reduce axonal dispersion and mistargeted regeneration [77]. Therefore, we believe that these measures of nerve conduit grafts prepared from rolled-up 2D submicron-grooved films or replicating submicron groove structures on NGC fillers could provide practical applications for peripheral nerve repair.

## 5. Conclusions

In our study, soft lithography is employed to fabricate submicron-grooved films with four different sizes. These films are utilized as cell culture platforms to assess the behaviors of Schwann cells. Our results reveal that submicron groove structures possess an efficacy similar to micron groove structures reported previously in directing the alignment and migration of Schwann cells. However, this effect is contingent on the depth of the submicron grooves. While the submicron groove structures significantly transform the morphology and cytoskeleton of Schwann cells, their impact on cell proliferation and cell cycle is negligible. More importantly, the submicron grooves can upregulate the gene expressions of Schwann cells responsible for axonal regeneration and myelination, which is promising for peripheral nerve repair. In sum, this study lays the groundwork for developing advanced biomaterials used for peripheral nerve repair.

## Figures and Tables

**Table 1:** The primer sequences used for RT-qPCR analysis.

Gene Name	Primer Sequences (5′→3′)
GAPDH	F: TCGGAGTCAACGGATTTGGT
	R: TTCCCGTTCTCAGCCTTGAC
Smad 6	F: GCTACCAACTCCCTCATCACT
	R: CGTACACCGCATAGAGGCG
MBP	F: CAGCACTAACCCTCCCTGAG
	R: GCTCAGCCCGTGTGTCTC
S100	F: CGGGGCACCCTTAAAACTTC
	R: ATCTGCATGGATGAGGAACGC
Sox10	F: CACAAGAAAGACCACCCGGA
	R: AAGTGGGCGCTCTTGTAGTG

## Data Availability

All the RNAseq raw data has been deposited at NCBI Gene Expression Omnibus (GEO) with the GEO accession number GSE226857.
